# Organization and evolution of the chalcone synthase gene family in bread wheat and relative species

**DOI:** 10.1186/s12863-019-0727-y

**Published:** 2019-03-18

**Authors:** Anastasia Y. Glagoleva, Nikita V. Ivanisenko, Elena K. Khlestkina

**Affiliations:** 1 Institute of Cytology and Genetics SB RAS, Novosibirsk, Russia; 2 Novosibirsk State University, Novosibirsk, Russia; 3 N.I. Vavilov All-Russian Research Institute of Plant Genetic Resources (VIR), Saint-Petersburg, Russia

**Keywords:** CHS, Chalcone synthase, Gene duplication, Flavonoid biosynthesis, Triticum, Aegilops

## Abstract

**Background:**

Flavonoid compounds are secondary plant metabolites, having a functional importance in plant development, protection from pathogens and unfavorable environmental factors. Chalcone synthase (CHS) is a key enzyme in the biosynthesis of flavonoids; it is involved in biosynthesis of all classes of flavonoid compounds. Nevertheless, the *Chs* gene family in bread wheat (*Triticum aestivum* L.) has been not characterized yet. The aim of the current study was to investigate structural and functional organization of the *Chs* genes and evolution of this gene family in bread wheat and relative species.

**Results:**

The nucleotide sequences of the eight *Chs* copies in *T. aestivum* were identified. Among them, two homoeologous sets of the *Chs* genes were located on the short (*Chs-A1*, *−B1*, *−D1*) and the long (*Chs-A4, −B4, −D4*) arms of homoeologous group 2 chromosomes. Two paralogous gene copies in the B-genome (*Chs-B2*, *−B3*) were located in the distal regions of 2BS chromosome. To clarify the origin of *Chs* duplications in the B-genome the phylogenetic analysis with the *Chs* sequences of *Triticum* and *Aegilops* species carrying ancestral genomes was conducted. It was estimated that the first duplication event occurred in the genome of the common ancestor of *Triticum* and *Aegilops* genera about 10–12 million years ago (MYA), then another copy was formed in the ancestor of the B-genome about 6–7 MYA. A homology modeling revealed high sequence similarity of bread wheat CHS enzymes. A number of short deletions in coding regions of some *Chs* sequences are not expected to have any significant functional effects. Estimation of transcriptional activity of the *Chs* copies along with a comparative analysis of their promoters structure suggested their functional specialization, which likely contributed to the maintaining of the duplicated *Chs* genes in wheat genome.

**Conclusions:**

From possible ten *Chs* copies in bread wheat genome, eight members of this family retained their intact structure and activity, while two copies appear to be lost at the level of diploid and tetraploid ancestors. Transcriptional assay along with a comparative analysis of the *cis*-regulatory elements revealed their functional diversification. The multiple functions supported by the *Chs* family are assumed to be a driving force for duplications of the *Chs* gene and their retention in plant genome.

**Electronic supplementary material:**

The online version of this article (10.1186/s12863-019-0727-y) contains supplementary material, which is available to authorized users.

## Background

Flavonoid compounds are secondary plant metabolites playing important role in plant development [1], protection against pathogens [2, 3] and environmental stresses such as UV light, drought, salinity, wounding, extreme temperatures [4, 5]. Flavonoids are antioxidants due to their ability to terminate free radical reactions and to protect the plants from oxidative stress [6]. Also, most of flavonoids are colored compounds, that made genes involved in their synthesis to be a convenient model for genetic studies [7]. The compounds are synthesized through the flavonoid biosynthesis pathway that starts from the condensation of three molecules of malonyl-CoA with one molecule of 4-coumaroyl-CoA with forming naringenin chalcone [8]. The reaction is catalyzed by chalcone synthase (CHS; EC 2.3.1.74), that belongs to polyketide synthases type III family.

The *Chs* genes are well-characterized in many plant species. Comparison of the *Chs* gene sequences from different species revealed that the *Chs* gene is structurally conserved, and most of the genes contain two exons and one intron. In all plant species, the CHS enzymes have the strictly conserved catalytic center consisted of four residues: Cys164, His303, Asn336, and Phe215 [9].

The *Chs* genes are generally represented in plant genomes by multiple copies forming gene families. Gene families can arise through gene duplications and subsequent nucleotide substitutions [10]. In some cases, duplicated genes increase the total amount of enzyme [11]. In other cases, gene family members can acquire new functions (neofunctionalization) [2] or can undergo the functional divergence establishing distinct complementary effects (subfunctionalization) [3, 12]. All cases were involved in plant adaptation to environments.

Bread wheat (*Triticum aestivum* L., *2n* = *6x* = 42, BBAADD) originated from different ancestral diploid species, related to nowadays existing *Triticum urartu* (*2n* = *2x* = 14, AA), *Aegilops speltoides* (*2n* = *2x* = 14, SS), and *Aegilops tauschii* (*2n* = *2x* = 14, DD) [13]. Because of the fusion of three genomes, most genes are present in three functional copies. They are referred to homoeologous genes. Non-additive gene expression of homoeologs from transcriptional divergence in different tissues or developmental stage up to epigenetic silencing has been widely reported [14–18].

In bread wheat, the *Chs* genes family has been not characterized yet. The aim of the current study was to investigate structural and functional organization of the *Chs* gene family and its evolution in bread wheat and relative species.

## Results

### The Chs gene copies identification and mapping

According to the known *Chs* gene sequence of barley *Hordeum vulgare* L. (X58339.1) the nucleotide sequences of the eight *Chs* copies in *T. aestivum* were identified in URGI database using a BLAST algorithm. The identified *Chs* copies were located on homoeologous group 2 chromosomes. Among them three homoeologous copies in the A-, B- and D-genomes (*Chs-A1, −B1* and *-D1*) and two paralogous copies in the B-genome (*Chs-B2*, *−B3*) were mapped using nulli-tetrasomic and deletion lines in the distal regions of 2AS, 2BS and 2DS chromosomes (Additional file 1). Three additional *Chs* copies (*Chs-A4*, *−B4*, *−D4*) were identified on the long arms of homoeologous group 2 chromosomes.

The *Chs* gene sequences of different *Triticum* and *Aegilops* species (tetraploid *T. durum* (*2n = 4x = 28*) and diploid *T. urartu*, *T. monococcum*, *Ae. speltoides*, *Ae. sharonensis*, and *Ae. tauschii* (*2n = 2x = 14*)) were also identified, annotated and used for phylogenetic analysis. The sequence of *Chs-A1* of *T. durum* was partially obtained by sequencing its PCR product. The identified copies are listed in Additional file 2: Table S2. Using FGENESH software the exon-intronic structure was determined: all *Chs* copies consist of two exons and one intron. The length of the coding sequence is 1185 bp. In sequences of several *Chs* copies (*Chs-A4 T. monococcum, Chs-A3, −A4 T. urartu*) the short deletions with frameshift in protein coding regions were observed.

### The origin of Chs duplicated copies

**Fig. 1:**
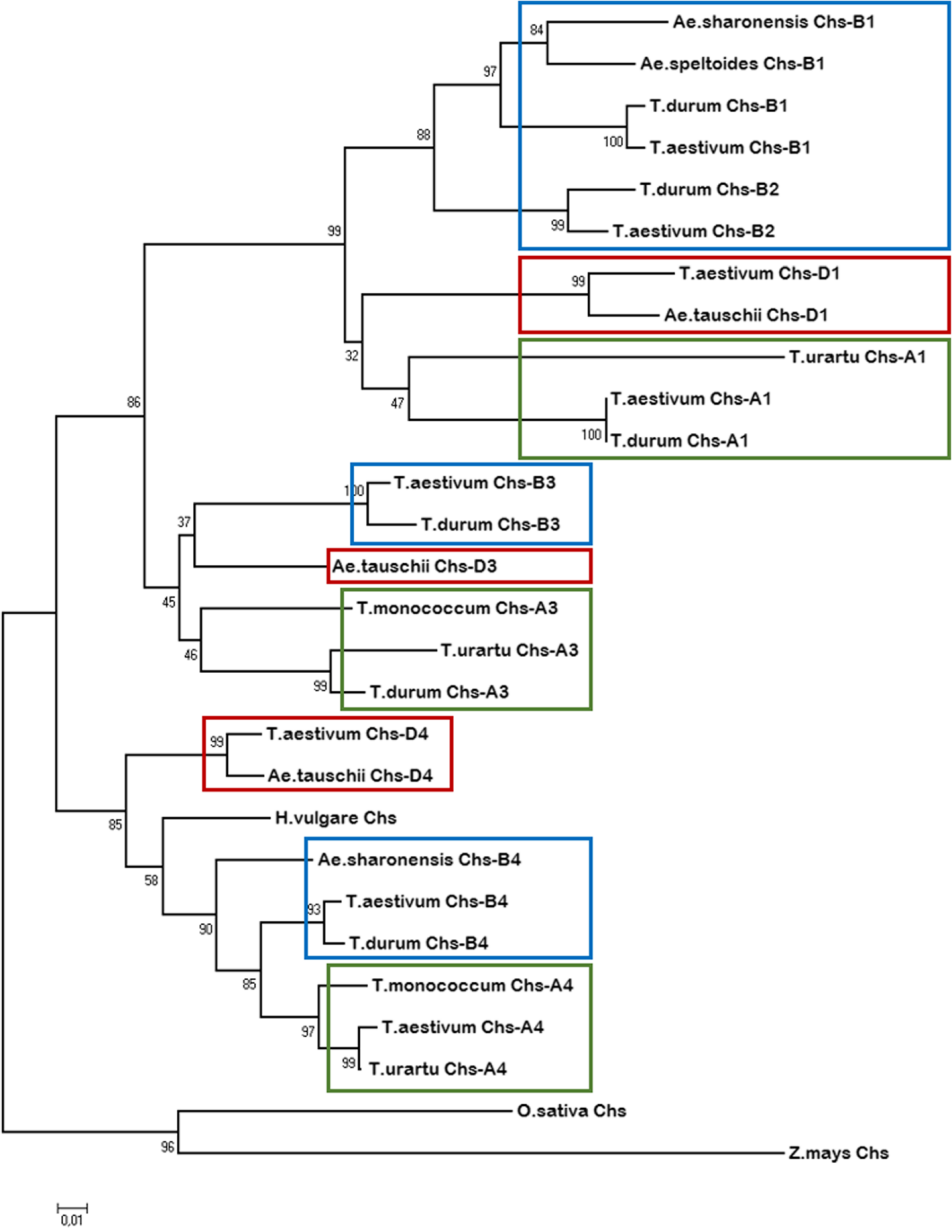
The Neighbour-Joining tree of the *Chs* genes of Triticeae species. The B/S-genomic copies are highlighted in blue frames, A-genomic – in green, D-genomic – in red

To clarify the origin of paralogous *Chs* duplications in the B-genome, we compared sequences of the *T. aestivum Chs* genes with their homologs in different *Triticum* and *Aegilops* species carrying the genomes A, B(S) and D. A phylogeny of the *Chs* copies is presented in Fig. 1. The *Chs* sequence of *Hordeum vulgare* (X58339.1), *Oryza sativa* (AB058397.1) and *Zea mays* (AY728478.1) were used as an outgroup.

For prediction the divergence time of the *Chs* copies in the B-genome, we used the number of synonymous substitutions between copies and the known time of divergence between *Hordeum* and *Triticum* (about 11 MYA). It was calculated that the first *Chs* gene duplication event leading to *Chi-B3* took place in the common ancestor of *Triticum* and *Aegilops* about 10–12 MYA. Then, the *Chi-B2* copy was formed in the ancestor of the B-genome about 6–7 MYA. The homoeologs (orthologs) of the *Chs-B3* copy are preserved in the A- and D-genomes of diploid and tetraploid species, but they were likely pseudogenized in *T. aestivum* 2A and 2D chromosomes. To confirm this assumption the primers specific for the *Chs-A3* sequences of *T. durum*, *T. monococcum* and *T. urartu* were designed. It was shown that the *Chs-A3* copy was preserved in diploid and tetraploid wheat species carrying A-genome, while in hexaploid wheat genomes this copy is absent (Table 1, Additional file 3). The *Chs-B2* copy does not have orthologs in the A and D genomes since it has occurred as the *Chs-B1* copy duplication in the ancestor of the B-genome (Fig. 1). The time of divergence between the *Chs* copies from 2S and 2 L chromosome lines is about 15 MYA.

**Table 1:** The results of the test of different *Triticum* species carrying A-genome for presence of the *Chs-A3* copy

Species	Genome	Number of samples	Expression
*Triticum aestivum* L.	BBAADD	4	no
*Triticum compactum* Host	BBAADD	1	no
*Triticum spelta* L.	BBAADD	1	no
*Triticum dicoccum* (Schrank) Schübl	BBAA	4	yes
		2	no
*Triticum dicoccoides* (Körn. ex Aschers. et Graebn.) Schweinf.	BBAA	3	yes
		3	no
*Triticum durum* Desf.	BBAA	1	no
*Triticum persicum* Vav.	BBAA	1	no
*Triticum timopheevii* Zhuk.	GGAA	1	yes
*Triticum urartu* Thum. Ex Gandil	AA	3	yes
*Aegilops speltoides* Tausch.	SS	3	no
*Aegilops tauschii* Coss.	DD	3	no

### Structural organization of the Chs copies

**Fig. 2:**
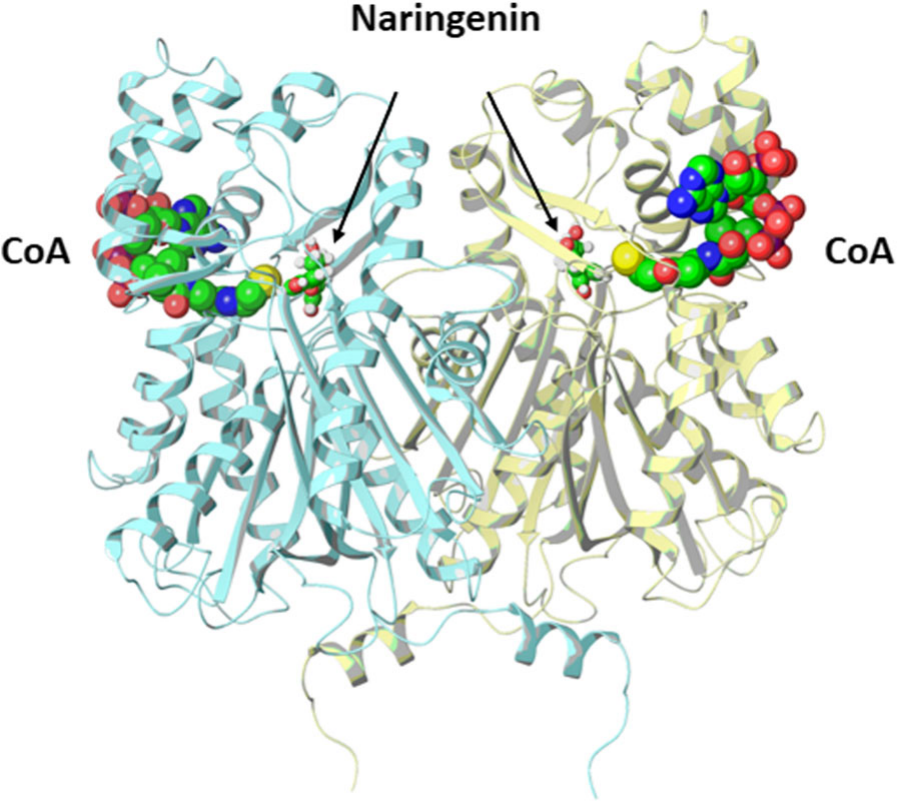
Homology model of CHS dimer in complex with CoA (CPK representation) and naringenin (ball and stick representation)

We used a homology modeling to analyze the effects of amino acid variability observed for *Chs* genes in bread wheat. All bread wheat CHS enzymes have high sequence similarity and more than 90% similarity with chalcone synthase from *Medicago sativa* L. with available crystal structures. The predicted homology model of CHS dimer enzyme in complex with CoA and naringenin are shown in Fig. 2. Presumably, all bread wheat predicted sequences encode functionally active chalcone synthases.

In some *Chs* sequences, we observed a number of short deletions in protein coding region (Additional file 4: Figure S1). The short deletion of one amino acid residue in *T. monococcum Chs-A4* sequence is located in the loop not involved directly in the catalytic activity of CHS, therefore we do not expect significant functional effects. Structural analysis of short deletion observed in *T. urartu Chs-A4* sequence showed that CHS can keep its function while binding specificity for CoA could be reduced due to loss of amino acid residues involved in recognition of phosphate group. Deletion observed in *T. urartu Chs-A3* leads to shifting of the active site loop and active site residue C167 (Additional file 4: Figure S1). This residue is reported as the active-site nucleophile in polyketide formation [19]. According to [19] substitution of this residue still keeps catalysis of malonyl-CoA decarboxylation by CHS without chalcone formation.

We conducted an analysis of amino acid sequence variability among CHS variants of protein using FoldX. As a result, most of amino acid substitutions were predicted to be neutral or stabilizing structure of the CHS. In addition, high sequence variability was observed for amino acid residues located close to CoA binding site, while in flavonoid binding site there were no amino acid substitutions. Two of the substitutions in question were shown to be highly destabilizing: A16E (*∆G*_*foldx*_ = 12.25 *kcal*/*mole*) and C63R (*∆G*_*foldx*_ = 12.6 *kcal*/*mole*) of *Ae. tauschii Chs-D1* and *T. monococcum Chs-A4* respectively. A16E is located on the N-terminal dimerization domain and therefore can be expected to reduce catalytic activity of CHS due to decrease of its dimerization propensity. C63 is located in hydrophobic region close to CoA binding site. According to FoldX, substitution to polar residue can lead to structural changes in CoA binding site. Indeed, we can expect that structural changes introduced by this substitution can lead to the formation of the salt bridge between R63 and D64 residues increasing its stability (Additional file 4: Figure S2).

### Functional diversification of the Chs copies

**Fig. 3:**
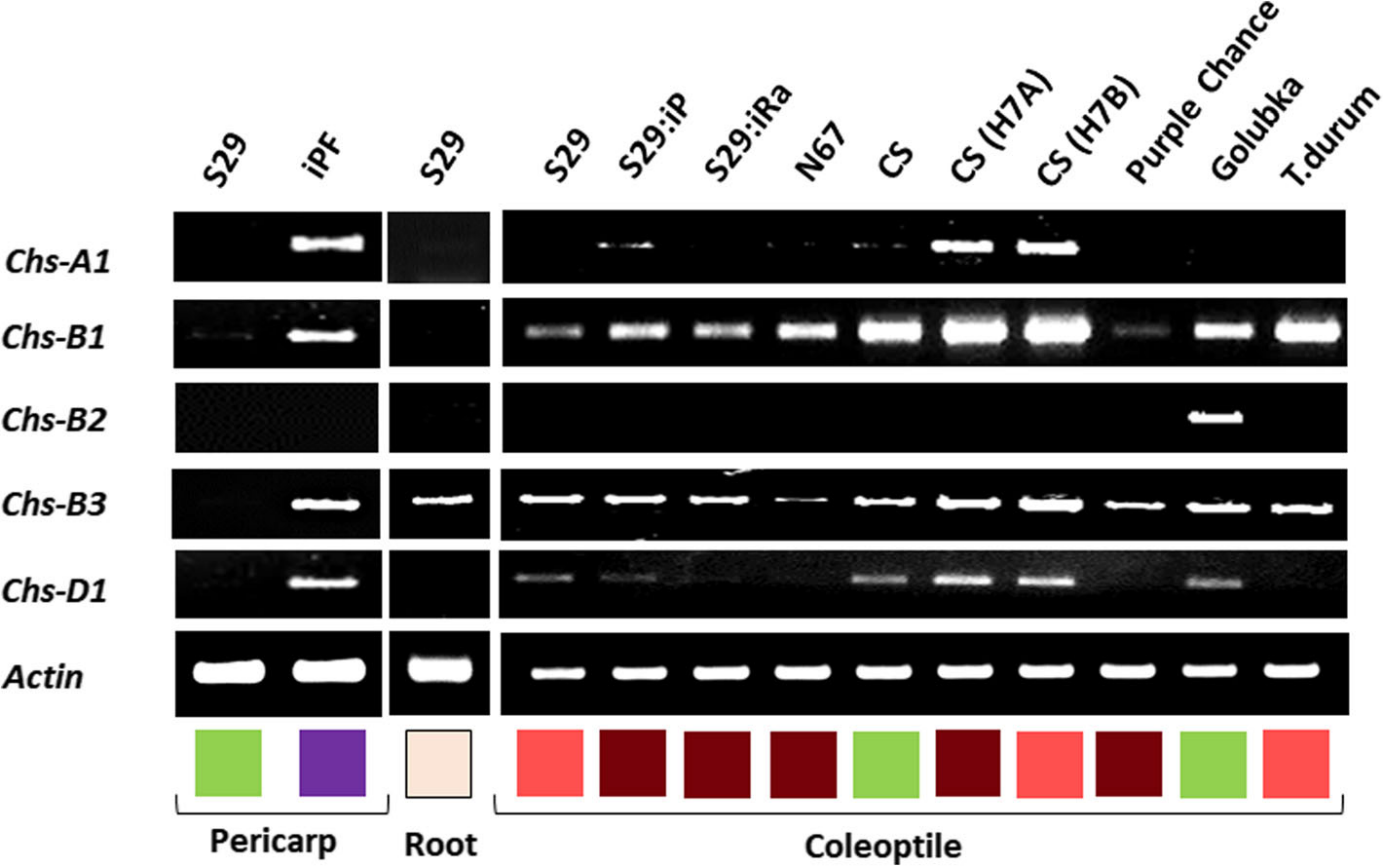
Expression of the *Chs* copies in the pericarps, roots and coleoptiles. In squares the color and pigmentation intensity of wheat tissues are shown. The lines and cultivars names: S29 - Saratovskaya 29, iPF - i:S29 Pp1Pp2^PF^ (PF - Purple Feed), iP - i:S29 Pp1Pp3^P^ (P – purple), iRa - i:S29Ra, N67 - Novosibirskaya 67, CS - Chinese Spring, CS(H7A) - Chinese Spring (Hope 7A), CS(H7B) - Chinese Spring (Hope 7B), *T.durum* – accession number TRI 15744

**Fig. 4:**
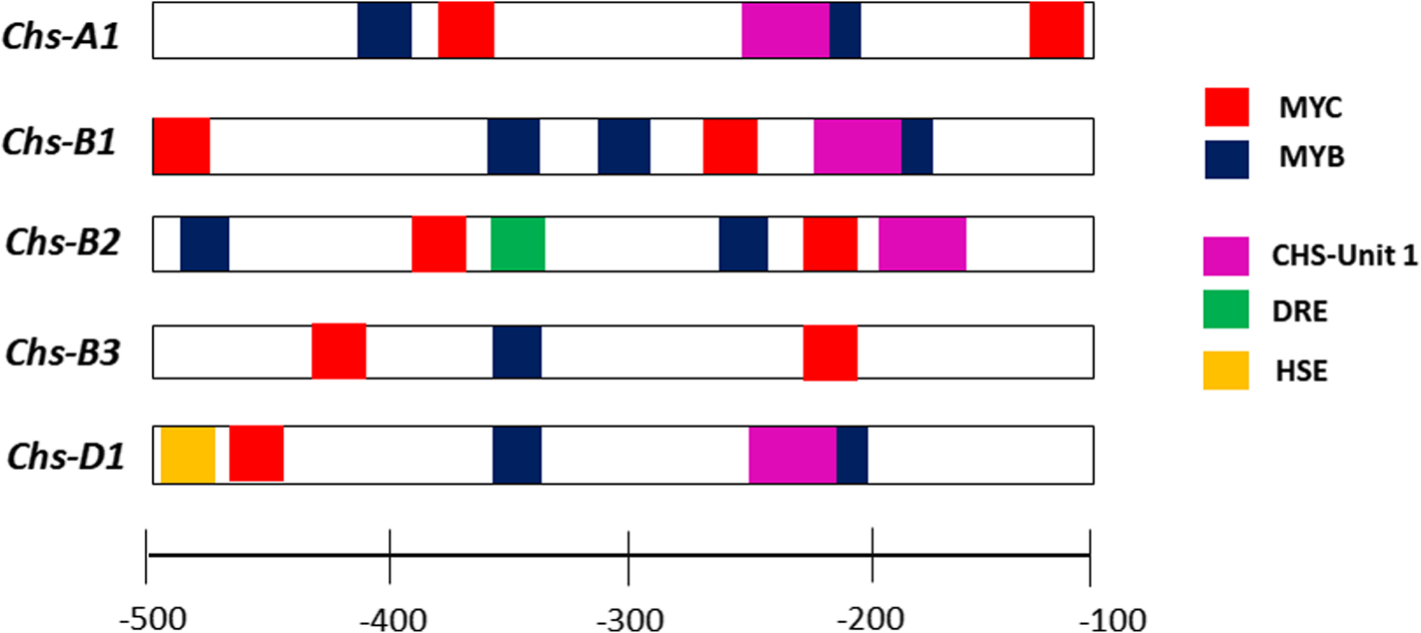
*Chs* promoter structure. *Cis*-regulatory elements: MYB and MYC binding sites, CHS-Unit 1 – light responsive element, DRE – cold- and dehydration-responsive element, HSE – heat stress responsive element

We compared expression of the *Chs-1, − 2, − 3* copies in different tissues: colored and uncolored pericarps, uncolored roots and coleoptiles with distinct pigmentation to test tissue specificity of the gene expression and dependence of their expression on the anthocyanin biosynthesis transcription factors. It was shown that all *Chs* copies are transcriptionally active, but they have different expression patterns (Fig. 3). All genes were active in the coleoptile, however, the genes were expressed independently on alleles of the *Rc* (*red coleoptile*) genes encoding MYB transcription factors, which define anthocyanin pigmentation and its intensity in coleoptiles. *Chs-B2* was expressed neither in the pericarp, nor in roots. It was active in the coleoptile of some genotypes only. *Chs-B3* was the only gene that is transcribed in roots. With the exception of *Chs-B2*, all genes were active in the pericarp, but the expression was dependent on alleles of the *Pp* (*purple pericarp*) genes encoding the MYB and MYC regulatory factors. The observed color-dependent regulation of the *Chs* genes confirms the functionality of the MYB and MYC transcription factors binding sites that were predicted in promoters of the *Chs* gene copies (Fig. 4). In addition to the MYB and MYC binding sites, the cold and dehydration response *cis*-element and heat stress response *cis*-element were predicted in the *Chs-B2* and *-D1* copies, respectively (Fig. 4). Overall, the data obtained demonstrated the functional divergence among the *Chs-1, − 2, − 3* gene copies in wheat. The *Chs-A4, −B4, −D4* copies were highly identical to each other that did not allow design copy-specific primers for testing their individual expression levels.

## Discussion

In this study, we identified the nucleotide sequences of the eight *Chs* genes in *T. aestivum*. All copies belong to class of chalcone synthases, but not to the other classes of polyketide synthases such as CHS-like or stilbene synthase (STS) [20]. All *Chs* copies were mapped to homoeologous group 2 chromosomes. It was shown that all copies have the same structure, consisting of two exons and one intron. The identified sequences showed the high level of coding sequences similarity (about 90%).

The duplicated genes can arise from ploidy events and/or segmental gene duplications. In allopolyploid wheat, both events can occur. Among *T. aestivum Chs* copies, we found two sets of homoeologous gene copies in the A-, B- and D-genomes (*Chs-A1, −B1*, *−D1* and *Chs-A4, −B4, −D4*) and two paralogous gene copies in the B-genome (*Chs-B2*, *−B3*). The paralogous gene copies *Chs-B2* and *Chs-B3* were originated because of segmental duplication. Thus, we found four copies of the *Chs* gene in B-genome of *T. aestivum*. For instance, in diploid genomes of dicot plants, the *Chs* gene family is usually represented by 6 to 12 members. In *Petunia hybrida*, eight complete and four partial *Chs* genes were identified [21], in poplar and the morning glory (*Ipomoea)*, the six *Chs* genes were found [2, 22]. In diploid genomes of monocots such as maize and rice, the 14 and 27 *Chs* genes were identified, respectively [23, 24].

It was calculated that the first *Chs* gene duplication event took place in the common ancestor of *Triticum* and *Aegilops* about 10–12 MYA, which led to the formation of *Chs-B3* copy. Then, another copy (*Chs-B2*) was formed in the ancestor of the B-genome about 6–7 MYA. Orthologs of the *Chs-B3* copy were preserved in the A- and D-genomes of diploid and tetraploid species, but the copies were likely pseudogenized in *T. aestivum* 2A and 2D chromosomes. Pseudogenization of duplicated gene copies in hexaploid wheat genome is widely occurring during polyploidization and domestication [25]. For instance, 11 of 17 predicted copies of the *Myc* gene were identified in *T. aestivum*, while six copies appear to be lost at the level of diploid ancestors [26]. It should be noted that a large number of wheat duplicated genes retain their functionality due to specialization [27, 28].The *Chs-B2* copy also does not have orthologs in the A and B genomes, since the duplication occurred in the ancestor of the B-genome. The *Chs* 2 L copies occurred in the ancestor of *Triticeae* tribe about 15 MYA.

It was shown that all identified *Chs* genes in bread wheat have different patterns of expression. All *Chs* genes with the exception of *Chs-B2* are related to anthocyanin biosynthesis in the pericarp, that is governed by the MYB- and MYC-responsive regulatory elements predicted in promoters of all copies. In coleoptiles, color-dependent regulation of the *Chs* copies was not observed. It can be explained by the different expression dynamics of the genes that dependent on genotypes. The *Chs-D1* gene is possibly involved in synthesis of flavonoid compounds during heat stress defense, which is related to the presence of the corresponding element. *Chs-B2* is not expressed in almost all genotypes, but unlike other copies, it has DRE elements in the promoter, associated with cold and drought resistance. *Chs-B2* probably activates synthesis of flavonoid compounds under stress conditions. Previously the role of the *Chs* gene in response to abiotic stresses such as wounding, low temperature, drought, salinity and UV-light has been reported [29]. For example, the expression level of *EaCHS1* from *Eupatorium adenophorum* in tobacco alters the accumulation of flavonoids and regulates plant tolerance to salinity stress by maintaining ROS homeostasis [30]. The accumulation of *Chs* mRNAs following wounding was observed in white spruce [31]. In other research the expression of the *Chs* gene in plants upon exposure to low temperature has been shown [32].*Chs-B3* gene is expressed in all tissues examined including roots. In promoter region of this gene, light responsive elements and part of MYB-recognition element are absent. Probably, it is required for constitutive expression of CHS for the synthesis of a wide range of flavonoids. Such specialization can be a reason for the maintenance of these duplicated genes in bread wheat genome.

## Conclusions

In the current study, we characterized the *Chs* genes family of bread wheat and its diploid and tetraploid relative species. Although some of the copies underwent pseudogenization, the identified eight *Chs* copies retained their intact structure and activity. Transcriptional assay along with a comparative analysis of the *cis*-regulatory elements revealed their functional diversification. The multiple functions supported by the *Chs* family are assumed to be a driving force for duplications of the *Chs* gene and their retention in the plant genome.

## Methods

### Plant material and DNA/RNA extraction

Samples of di-, tetra-, and hexaploid wheat species as well as *Aegilops* species maintained in collections of VIR, IPK and ICG used in the current study are listed in Additional file 2: Table S1. In addition, we used the complete set of cv. Chinese Spring nulli-tetrasomic lines [33] and a set of homoeologous group 2 chromosomes deletion lines [34] for chromosomal and intra-chromosomal localization of the wheat *Chs* genes. DNA was extracted from the seven-day-old seedlings following the procedure described by Plaschke et al. [35]. The other genetic stocks are listed in Additional file 2: Table S1.

RNA was extracted from four-day-old coleoptiles, roots, colored and uncolored pericarp (early dough stage maturity, BBCH code 83) using QIAGEN Plant RNeasy kit (Hilden, Germany) followed by DNAse treatment applying RNase-Free DNase Set (QIAGEN, Hilden, Germany). For RNA extraction the seedlings were grown in climatic chamber «Rubarth Apparate» (RUMED GmbH) at 20 °C under a 12 h photoperiod. For RNA extraction from pericarp plants were grown at the Institute of Cytology and Genetics Greenhouse Core Facilities (Novosibirsk, Russia) under 12 h of light per day at 20–25 °C.

### Identification of wheat Chs gene copies and sequence analysis

Wheat *Chs* gene sequences search was performed using BLAST algorithm in three databases (https://urgi.versailles.inra.fr/blast/blast.php, http://www.cerealsdb.uk.net/cerealgenomics/CerealsDB/search_reads.php, www.ncbi.nlm.nih.gov/Database/) within genomic sequences of *T. aestivum* and its tetraploid (*T. durum*) and diploid (*T. monococcum, T. urartu, Ae. speltoides, Ae. sharonensis* and *Ae. tauschii*) relatives. For the identified *Chs* copies of *T. aestivum*, the set of copy-specific primers was designed using IDT PrimerQuest software (http://eu.idtdna.com/PrimerQuest/Home/) (Table 2). The sequence of *T. durum Chs-A1* copy was partially obtained using ABI PRISM 310 Genetic Analyzer (Perkin Elmer Cetus). Gene sequences were annotated using FGENESH software (Softberry Inc.,linux1.softberry.com/berry.phtml). The multiple alignment of gene sequences was performed using Multaline software [36]. Phylogenetic tree was constructed using MEGA 7 software [37] in conjunction with the Neighbor-Joining algorithm and 1000 bootstrap replicates. The number of synonymous (Ks) and non-synonymous (Ka) substitutions was evaluated following by Nei and Gojobori [38] method. The *cis*-elements in promoters were predicted using PlantCARE software [39], The transcription start site was determined by the TSSP program (Softberry Inc., linux1.softberry.com/berry.phtml).

**Table 2:** *Chs* copy-specific primers designed in the current study

Purpose	Name	Sequence	Amplicon lengh (bp)	Annealing temperature (°C)
Copy identification, chromosomal assignment and mapping, RT-PCR	Chs-A1	Forward 5’TTGTATTCTGCACCACCTCG3’	265	62
		Reverse 5’AAGAGTGCCTGACCTACCA3’		62
	Chs-B1	Forward 5’TCGTCGTCGTGGAGGTT3’	256	56
		Reverse 5’TCGGAGCAGACCACCA3’		56
	Chs-B2	Forward 5’ATGTATCAGCAGGGCTGT3’	279	54
		Reverse 5’CTCTGAGTCCGGCAGAAT3’		54
	Chs-B3	Forward 5’GAAGAGGTACATGCACCTG3’	481	53
		Reverse 5’CGCACCGACGATCACG3’		57
	Chs-D1	Forward 5’GATCACCCACCTTGTATT3’	257	49
		Reverse 5’CGCCTGACCTACCAGT3’		54
Chs-A3 group-specific	Chs_gs_A3	Forward 5’CCCACCTCGACTCGCTA3’	409	56
		Reverse 5’GCACTTGACATGTTGCCATATT3’		54
Sequensing	T.durum_A1	Forward_1 5’AGGAAGAGGTACATGCACCTT3’	567	56
		Reverse_1 5’GATGGCACCCTCTGAGTCT3’		56
		Forward_2 5’CGCTGGTAGGTCAGGCA3’	543	57
		Reverse_2 5’CTGGGACACTATGGAGGACAA3’		56
Reference	HvActin	Forward 5’TCGCAACTTAGAAGCACTTCCG3’	130	60
		Reverse 5’AAGTACAGTGTCTGGATTGGAGGG3’		60

### Protein structure analysis

Homology modeling was carried out using Modeller 9v12 [40]. Sequence alignments were obtained by Clustal-O program [41]. Model with the best DOPE score among 1000 generated models was selected. Crystal structure of chalcone synthase from *Medicago sativa* was used as the template (PDB identifier 1D6H; [19]). Crystal structure of CHS in complex with CoA and naringenin was obtained by structural superimposition with CHS/CoA (PDB identifier 1D6H) and CHS/Naringenin complexes (PDB identifier 1CKG; [9]). Estimations of impact of amino acid substitutions on stability were done using FoldX software [42]. The preliminary homology model was optimized using FoldX RepairPDB module.

### Chromosomal assignment of the wheat Chs gene copies and Chs-A3 copy searching

PCR amplification from genomic DNA template of the cv. Chinese Spring nulli-tetrasomic and deletion lines was performed in 20 μl reactions each containing 1 U Taq DNA polymerase (Medigen Ltd.), 1x PCR buffer (67 mM TrisHCl, pH 8.8; 18 mM (NH_4_)_2_SO_4_; 0.01% Tween 20), 0.2 mM dNTP and 0.25 μM of each primers. 1.5 or 1.8 mM MgCl_2_ was used for amplification of *Chs-A1*, *−B3* and *Chs-B1*, *−B2*, −*D1*, *Chs-gs-A3* respectively. The amplification regime was initiated by a denaturing step (94 °C/2 min), followed by 13 cycles of 94 °C/15 s, 65 °C/30 s with the temperature decrease in each cycle on 0,7 °C, 72 °C/45 s, when 24 cycles of 94 °C/45 s, 56 °C/30s, 72 °C/45 s and completed with a final extension step of 72 °C/5 min.

### RT-PCR

Single-stranded cDNA was synthesized from 1 μg total RNA using a (dT)_15_ primer and a RevertAid™ kit (Thermo Fisher Scientific Inc., Waltham, MA, USA) in a 20 μl reaction. RT-PCRs were conducted with the *Chs* copy-specific primers with the amplification regime and conditions describing above. The *Actin* gene sequence was used as a reference [43].

## Additional files

Additional file 1: Intra-chromosomal localization of the *Chs* genes within the homoeologous group 2 chromosomes derived from the analysis of PCR profiles of deletion lines. (PDF 187 kb)

Additional file 2: **Table S1.** Wheat cultivars and accessions of *Triticeae* species using for PCR searching of Chs-A3 copy. **Table S2.** URGI contig numbers of identified *Triticum* and *Aegilops* sequences. (DOCX 21 kb)

Additional file 3: The PCR profile of *Chs-A3* copy searching in different *Triticum* and *Aegilops* species. (PNG 37 kb)

Additional file 4: **Figure S1.** The illustration of the impact of short deletions to CHS protein structure. **Figure S2.** Analysis of amino acid substitutions in the wheat CHS sequence and estimation of impact of amino acid substitutions on protein stability. (DOCX 1111 kb)

## Abbreviations

MYA: million years ego
MYB: myeloblastosis
MYC: myelocytomatosis
*Pp*: Purple pericarp
*Rc*: Red coleoptile
