# Supplementary figures and images for: Organization and evolution of the chalcone synthase gene family in bread wheat and relative species
Source: BMC Genet. 2019 Mar 18;20(Suppl 1):30. doi: 10.1186/s12863-019-0727-y (PMC6421938; doi:10.1186/s12863-019-0727-y)

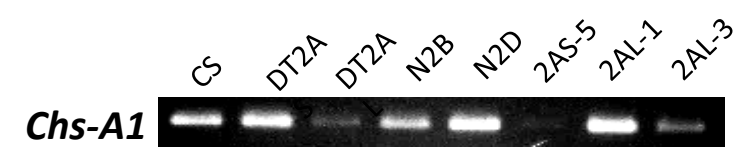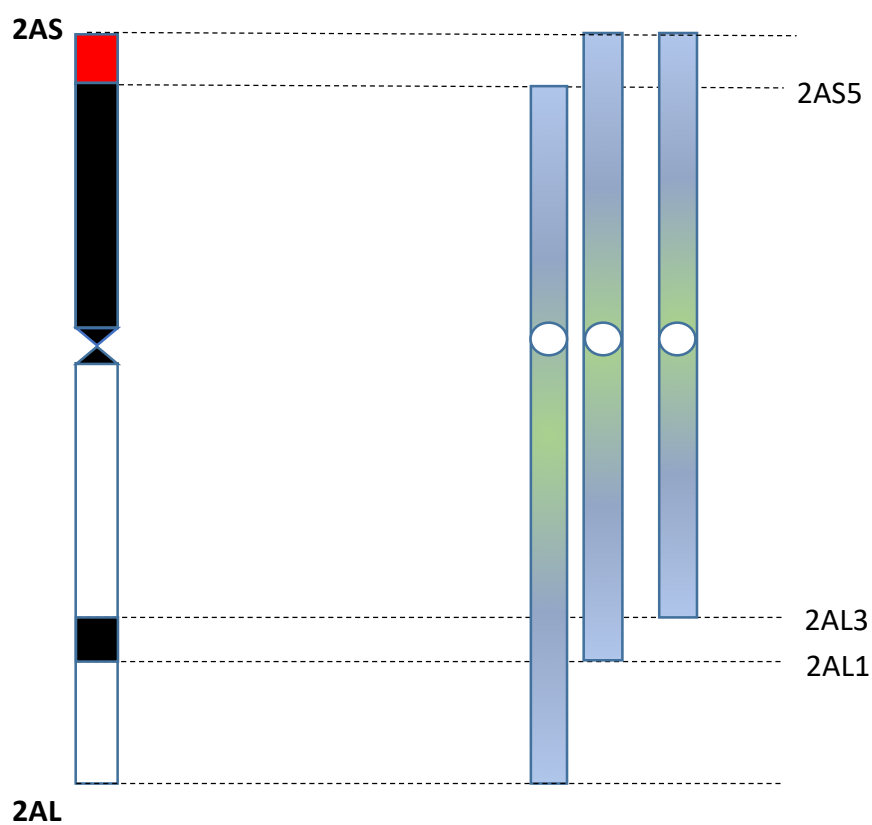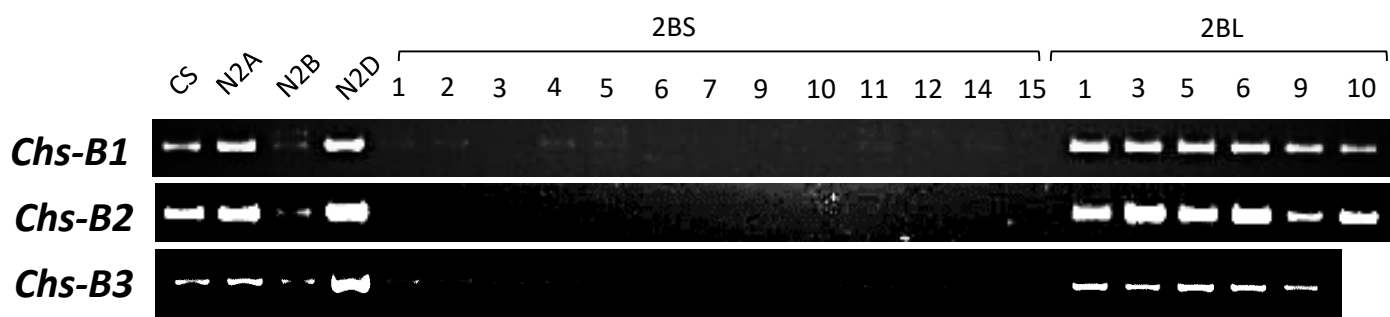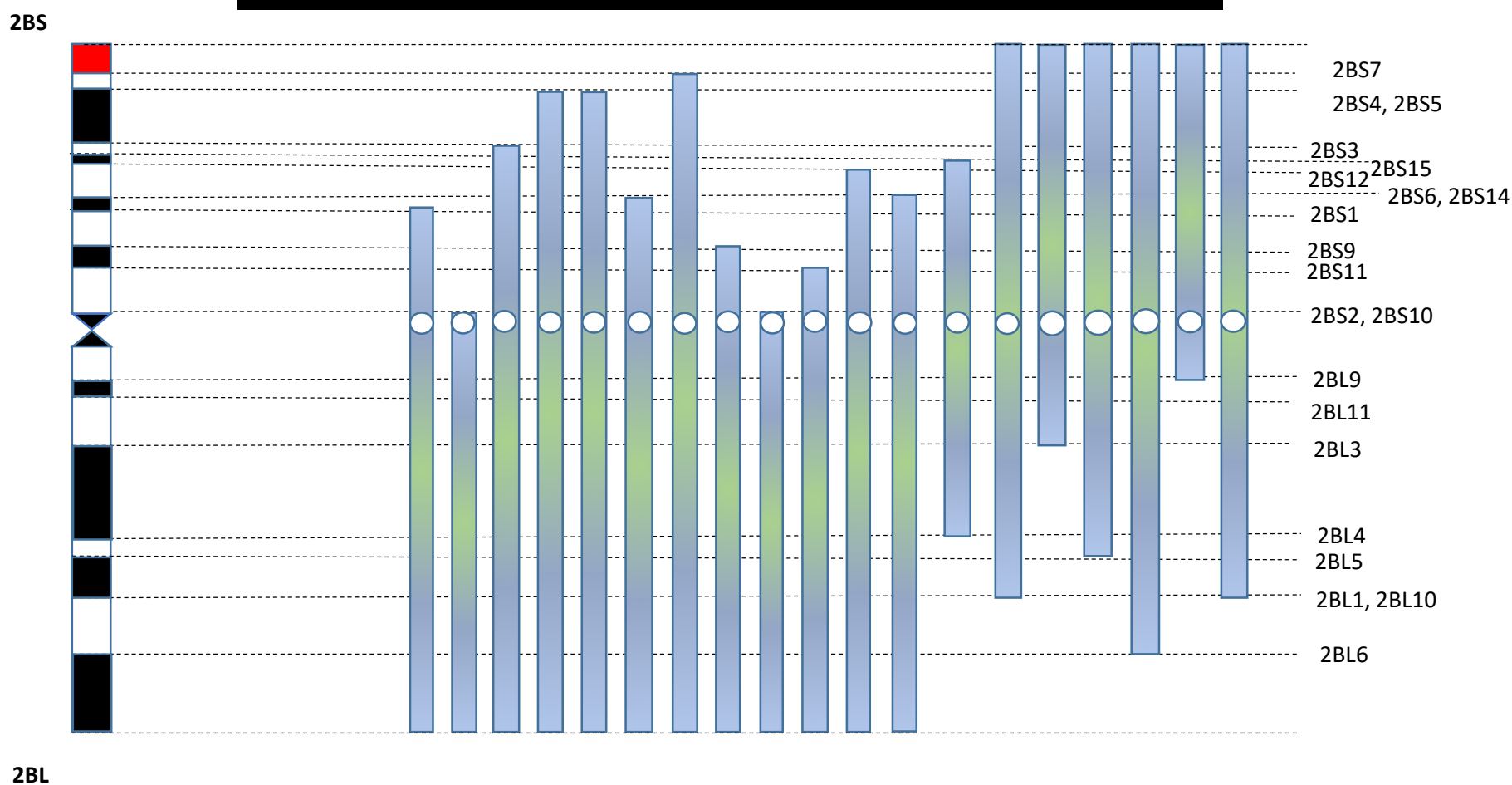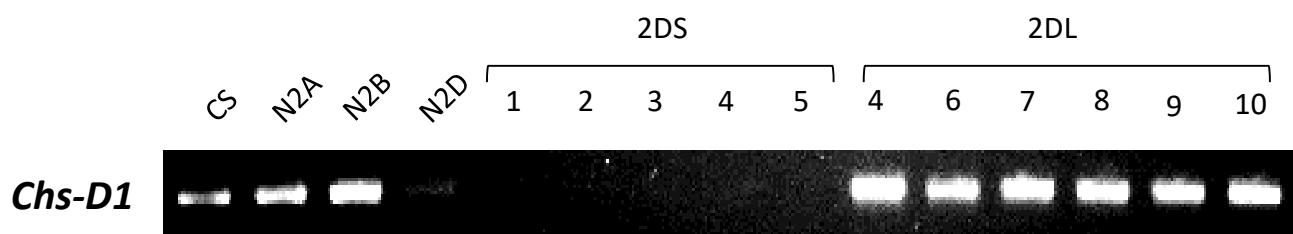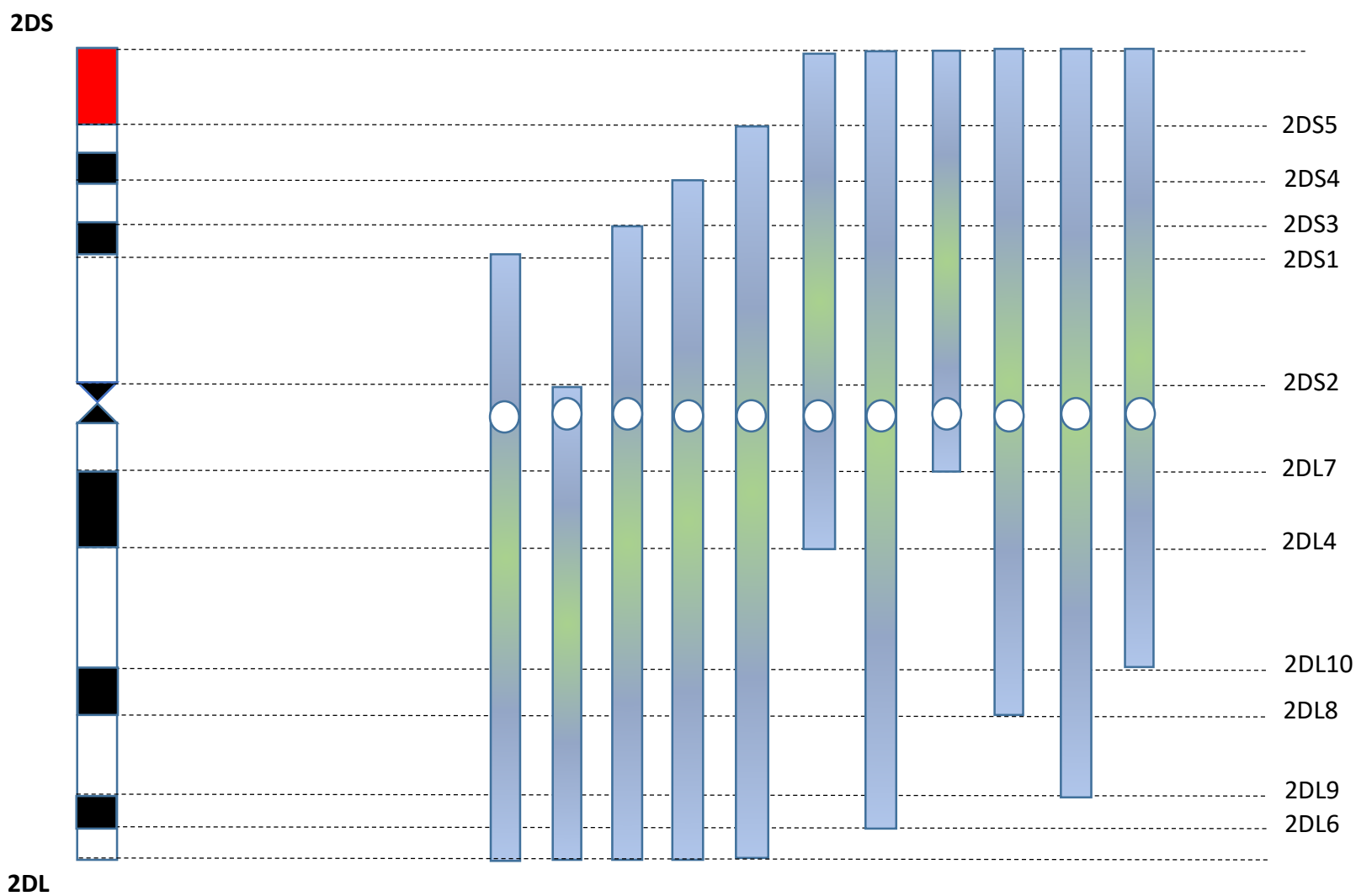

Supplement: Supplementary file 1 — Intra-chromosomal localization of the Chs genes within the homoeologous group 2 chromosomes derived from the analysis of PCR profiles of deletion lines. (PDF 187 kb) [file 12863_2019_727_MOESM1_ESM.pdf]

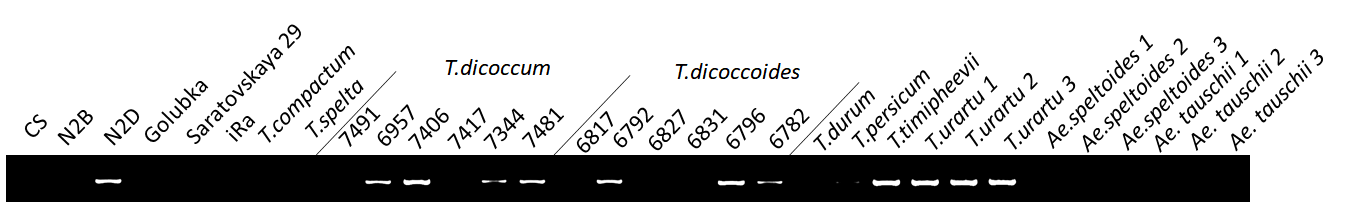

Supplement: Supplementary file 3 — The PCR profile of Chs-A3 copy searching in different Triticum and Aegilops species. (PNG 37 kb) [file 12863_2019_727_MOESM3_ESM.png]
