# Supplementary material for: Organization and evolution of the chalcone synthase gene family in bread wheat and relative species
Source: BMC Genet. 2019 Mar 18;20(Suppl 1):30. doi: 10.1186/s12863-019-0727-y (PMC6421938; doi:10.1186/s12863-019-0727-y)
Supplement: Supplementary file 2 — Table S1. Wheat cultivars and accessions of Triticeae species using for PCR searching of Chs-A3 copy. Table S2. URGI contig numbers of identified Triticum and Aegilops sequences. (DOCX 21 kb) [file 12863_2019_727_MOESM2_ESM.docx]

**Table S1.** Wheat cultivars and accessions of Triticeae species used for PCR searching of *Chs-A3* copy.

| **Genome** | **Species** | **Sample/No/Var.** |
| --- | --- | --- |
| BBAADD (2n=6x=42) | *Triticum aestivum* L. | Chinese Spring |
|  |  | Golubka |
|  |  | Saratovskaya 29 (S29) |
|  |  | i:S29Ra |
|  | *Triticum compactum* Host | k-221180^2^ |
|  | *Triticum spelta* L. | k-53660^2^ |
| BBAA (2n=4x=28) | *Triticum dicoccoides* (Körn. ex Aschers. et Graebn.) Schweinf. | 6817^1^ |
|  |  | 6792-1^1^ |
|  |  | 6827^1^ |
|  |  | 6817^1^ |
|  |  | 6792-1^1^ |
|  |  | 6827^1^ |
|  | *Triticum dicoccum* (Schrank) Schübl. | 6817^1^ |
|  |  | 6792-1^1^ |
|  |  | 6827^1^ |
|  |  | 6817^1^ |
|  |  | 6792-1^1^ |
|  |  | 6827^1^ |
|  | *Triticum durum* Desf. | TRI 2719^3^ |
|  | *Triticum persicum* Vav. | k-13383^2^ |
| GGAA (2n=4x=28) | *Triticum timopheevii* Zhuk. | 38555 |
| AA (2n=2x=14) | *Triticum urartu* Thum. Ex Gandil |  |
| SS (2n=2x=14) | *Aegilops speltoides* Tausch. |  |
| DD (2n=2x=14) | *Aegilops tauschii* Coss. |  |

^1^ Accession number in the collection of the Institute of Cytology and Genetics (Novosibirsk, Russia).

^2^ Accession number in the collection of the Vavilov Research Institute for Plant Industry (St. Petersburg, Russia).

^3^ Accession number in the collection of the Leibniz Institute of Plant Genetics and Crop Plant Research (Gatersleben, Germany).

**Table S2.** URGI contig numbers of identified *Triticum* and *Aegilops* sequences.

| **Species** | **Copy name** | **URGI contig number** |
| --- | --- | --- |
| *Triticum aestivum* L*.* | TaChs-A1 | 5222811 |
|  | TaChs-B1 | 5229518 |
|  | TaChs-B2 | 5244189 |
|  | TaChs-B3 | 5201747 |
|  | TaChs-D1 | 5335048 |
|  | TaChs-A4 | 6353002 |
|  | TaChs-B4 | 8050258 |
|  | TaChs-D4 | 9842237 |
| *Triticum durum* Desf. | TdChs-B1 | 158710 |
|  | TdChs-B2 | 17686 |
|  | TdChs-B3 | 2295470 |
|  | TdChs-B4 | 378077 |
|  | TdChs-A1 | GenBank:MH704854 |
|  | TdChs-A3 | 664678 |
| *Triticum urartu* Thum. Ex Gandil | TuChs-A1 | 189017 |
|  | TuChs-A3 | 182740 |
|  | TuChs-A4 | 320098+1357855 |
| *Triticum monococcum* L. | TmChs-A3 | 918992 |
|  | TmChs-A4 | 2202976 |
| *Aegilops speltoides* Tausch. | Ae.spChs-B1 | 198147 |
| *Aegilops sharonensis* Eig. | Ae.shChs-B1 | 89655 |
|  | Ae.shChs-B4 | 91289 |
| *Aegilops tauschii* Coss. | Ae.tChs-D1 | 106569 |
|  | Ae.tChs-D3 | 103322 |
|  | Ae.tChs-D4 | 162223+190020 |
