# Supplementary material for: Organization and evolution of the chalcone synthase gene family in bread wheat and relative species
Source: BMC Genet. 2019 Mar 18;20(Suppl 1):30. doi: 10.1186/s12863-019-0727-y (PMC6421938; doi:10.1186/s12863-019-0727-y)

**Figure S1.** The illustration of the impact of short deletions to CHS protein structure. Structural superimposition of homology models of the reference CHS structure (blue color) and target structures (purple color) are shown. Short deletions in *T. urartu Chs-A3* (**A**) and *T. urartu Chs-A4* (**B**) sequences can lead to structural changes in the catalytic site and CoA binding sites respectively. Short deletions in *T. monococcum Chs-A4* (**C**) is expected to be neutral. Amino acid residues are shown in ball and stick representation in gray color for reference structure and black color for target structure.


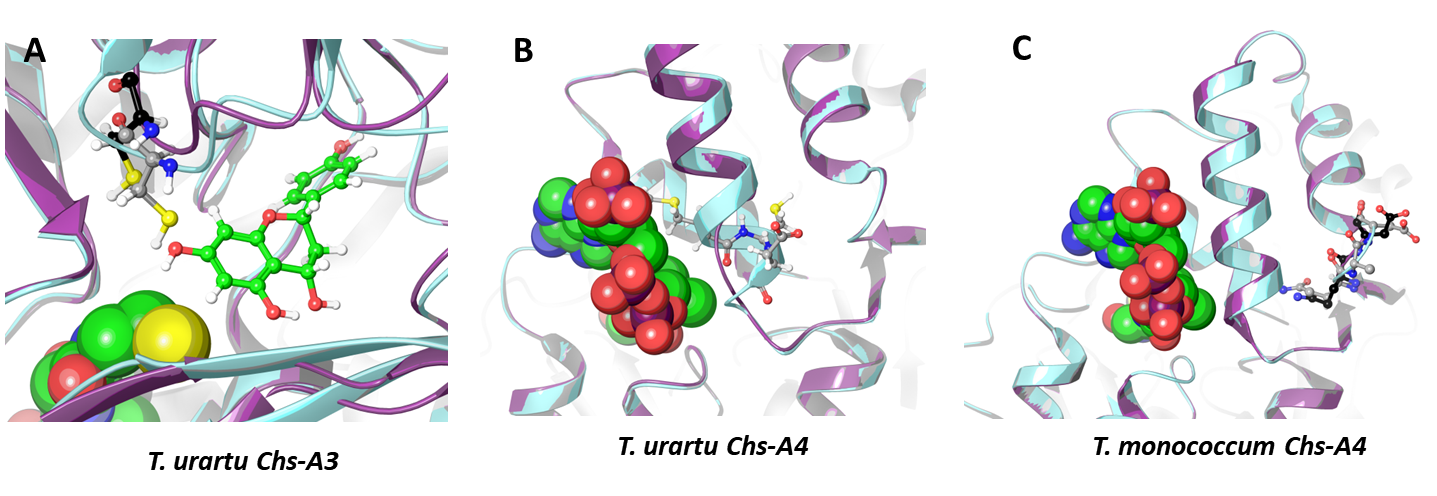


**Figure S2**. Analysis of amino acid substitutions in the wheat CHS sequence. (**A**) Estimation of impact of amino acid substitutions on protein stability using FoldX. Blue color gradient indicates increase in stability, red color gradient – decrease in stability. Two amino acid substitutions with highest predicted impact on stability are highlighted. (**B**) Detailed view of destabilizing C63R substitutions in *T. monococcum Chs-A4* sequence. R63 is located close to D64 and expected to form salt bridge.


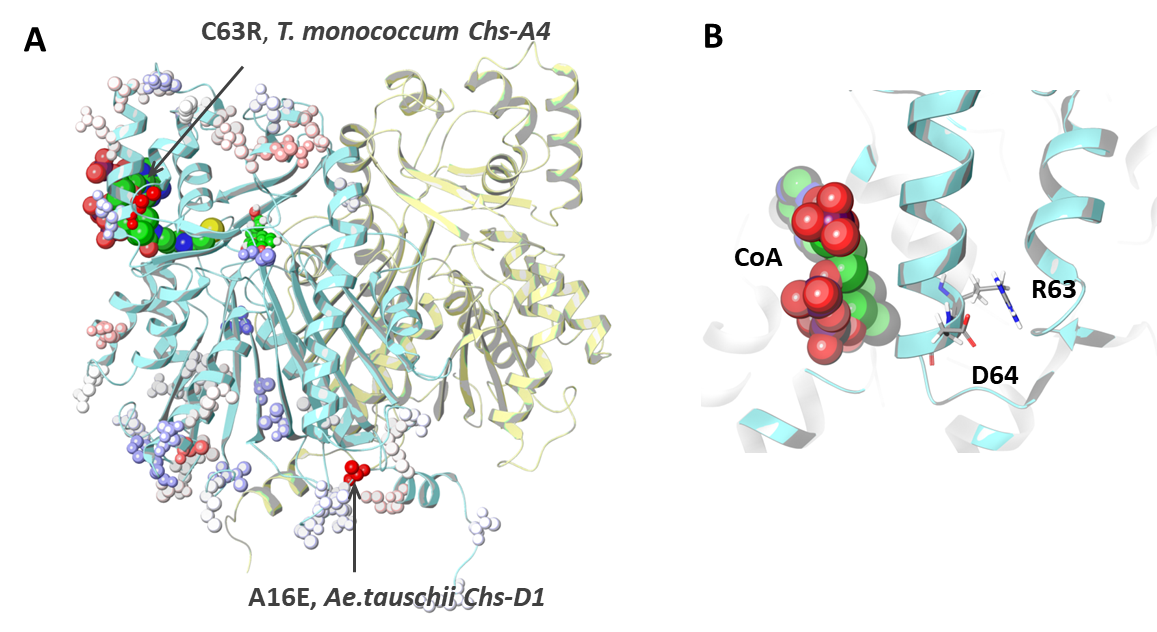

Supplement: Supplementary file 4 — Figure S1. The illustration of the impact of short deletions to CHS protein structure. Figure S2. Analysis of amino acid substitutions in the wheat CHS sequence and estimation of impact of amino acid substitutions on protein stability. (DOCX 1111 kb) [file 12863_2019_727_MOESM4_ESM.docx]
